# VIRTUAL RESPITE FOR FAMILY CAREGIVERS

**DOI:** 10.1093/geroni/igad104.1752

**Published:** 2023-12-21

**Authors:** Tiffany Washington, Jennifer Rapowitz

**Affiliations:** University of Georgia, Athens, Georgia, United States; University of Georgia, Athens, Georgia, United States

## Abstract

The COVID-19 pandemic was detrimental to the wellbeing of individuals everywhere, especially family caregivers. Over 20% of older caregivers reported difficulties finding time for self-care during the pandemic Respite is an avenue to self-care; however, virtual respite programs are nearly non-existent. In this proof of concept study, we examined the acceptability of a virtual respite program with eight family caregivers of individuals living with dementia. Virtual respite visits were delivered by graduate students who engaged the person with dementia in a tailored activity, thereby freeing time for the caregiver to have a respite break. Families received up to six weekly virtual respite visits totaling 122 to 295 minutes. Semi-structured interviews with participating caregivers were transcribed and coded using steps of thematic analysis. Caregivers expressed high acceptability for virtual respite because it was easily adaptable to their existing hardware (e.g., smart devices, laptops), accessible, and required little technological knowledge. They further expressed appreciation for tailored activities for care receivers that allowed respite time for their own self-care activity. Given the limited availability of respite services, there is a need to design and test new types of respite care. This virtual respite program is an opportunity to reach more caregivers.

